# Nanoformulation improves antitumor efficacy of MAOI immune checkpoint blockade therapy without causing aggression-related side effects

**DOI:** 10.3389/fphar.2022.970324

**Published:** 2022-09-01

**Authors:** James Brown, Zhe Li, Xi Wang, Yu Jeong Kim, Yu-Chen Wang, Yanning Zuo, Weizhe Hong, Pin Wang, Bo Li, Lili Yang

**Affiliations:** ^1^ Department of Microbiology, Immunology and Molecular Genetics, University of California, Los Angeles, CA, United States; ^2^ Department of Biological Chemistry, David Geffen School of Medicine, University of California, Los Angeles, CA, United States; ^3^ Department of Neurobiology, David Geffen School of Medicine, University of California, Los Angeles, CA, United States; ^4^ Department of Pharmacology and Pharmaceutical Sciences, University of Southern California, Los Angeles, CA, United States; ^5^ Eli and Edythe Broad Center of Regenerative Medicine and Stem Cell Research, University of California, Los Angeles, CA, United States; ^6^ Jonsson Comprehensive Cancer Center, The David Geffen School of Medicine, University of California, Los Angeles, CA, United States; ^7^ Molecular Biology Institute, University of California, Los Angeles, CA, United States

**Keywords:** monoamine oxidase inhibitors, phenelzine, immune checkpoint blockade, nanoformulation, cancer immunotherapy, neurological side effects, crosslinked multilamellar liposomal vesicles, serotonin

## Abstract

MAOIs, a well-established class of antidepressant that operate through the inhibition of monoamine oxidase to increase available serotonin, have recently been identified as a surprisingly effective candidate for the circumvention of tumor-induced immune suppression due to their abilities to enhance antitumor T cell activity through autocrine serotonin signaling and depolarize alternatively activated tumor-associated macrophages through a reduction in reactive oxygen species production. However, this impressive class of antidepressants-turned-cancer-drugs can induce aggressive behavioral side effects when administered in immunotherapeutic doses. In this study, we investigated the possibility of avoiding these neurological side effects while simultaneously improving antitumor activity by establishing crosslinked multilamellar liposomal vesicles (cMLVs) containing the MAOI phenelzine (PLZ). Our results showed that cMLV-PLZ treatment increases antitumor efficacy in a B16-OVA mouse melanoma model compared to treatment with free phenelzine. We also found that nanoformulation resulted in the complete elimination of MAOI-related aggression. These findings suggest a promising direction for the future of MAOIs repurposed for cancer immunotherapies.

## Introduction

The global effort to cure cancer has seen tremendous developments in the past few decades, with novel technologies and approaches giving rise to entirely new fields of cancer therapeutics (Atlihan-Gundogdu et al., 2020). One field that has seen impressive growth in recent years is immunotherapy, which seeks to rid the body of tumor cells through the artificial augmentation of the human immune system’s natural antitumor abilities (Chen and Mellman, 2013; Vermaelen et al., 2018; Brown et al., 2022). Tumors are incredibly adaptive due to their lack of regulation on gene replication (Guan et al., 2022), which can result in the development of impressive anti-inflammatory conditioning within the tumor microenvironment (TME) to promote tumor growth and exploit the inhibitory checkpoints that exist to regulate the immune system under normal conditions (Cassim and Pouyssegur, 2020). Because of the immunosuppressive nature of the TME, tumors are often able to eliminate one of the body’s best defenses against tumors: cytotoxic T cells (Ngiow et al., 2015; YeonYeon et al., 2019). However, due to advances in immunotherapy, the field of immune checkpoint blockade (ICB) has arisen as a means of circumventing the tumor-induced stimulation of inhibitory checkpoints. Leading therapies include anti-PD-1/anti-PD-L1 antibody therapy, which blocks the stimulation of programmed death receptor 1 (PD-1) on T cells, and anti-CTLA4, which blocks the inhibition of T cell function by antagonizing Cytotoxic T Lymphocyte-associated Antigen 4 (CTLA4) (Littman, 2015; Wang et al., 2019). However, tumor type and T-cell phenotype can greatly impact the efficacy of these drugs, resulting in their failure in many patients (YeonYeon et al., 2019). Despite their shortcomings in many patients, the successes of these drugs have inspired the search for novel ICB candidates for mono- and combination therapies that target multiple different cell types and use diverse methods of bypassing the immunosuppressive nature of the TME (Brown et al., 2022).

MAOIs are a class of small-molecule drug that inhibit the enzyme monoamine oxidase-A (MAO-A), which was first discovered in the brain as the regulatory degrader of monoamine neurotransmitters such as serotonin and dopamine (Pintar and Breakefield, 1982). Due to their inhibition of serotonin depletion, MAOIs were first approved for treatment of clinical depression in the 1950s and have since gone on to treat a range of mental ailments from Alzheimer’s to Parkinson’s (Bortolato et al., 2008; Carradori et al., 2018). The inhibition of MAO-A increases serotonin available for neurotransmission, subsequently increasing the incidence of 5HTR agonism in the postsynaptic neuron and mitigating the effects of clinical depression (Pintar and Breakefield, 1982).

However, despite the efficacy of MAOIs as antidepressants, they have fallen largely out of fashion as a first-line prescription with the rise in popularity of newer drugs and misconceptions surrounding the severity of MAOI-related side effects (Volz and Gleiter, 1998; Gillman, 2018). In high doses or combination with other serotonergic drugs, MAOIs have been linked to serotonin syndrome, which results from an overstimulation of the serotonin receptor and causes neuronal and physical maladies such as delirium and neuromuscular hyperactivity. In the past, severe hypertensive side effects due to an overaccumulation of tyramine, which can result from the consumption of tyramine-rich foods such as cheese and turkey, known as “the cheese effect” were sometimes reported in patients taking MAOIs (Raghupathi et al., 2013; El-Merahbi et al., 2015; Gillman, 2018). However, modern food production practices have greatly limited the diet-associated risks of MAOIs, improving their safety profile (Gillman, 2018). These side effects have affected the marketability of MAOIs as antidepressants and have confined them to primarily being used for the treatment of more severe neuronal disorders such as Parkinson’s. However, as MAOIs and the MAO enzymes become increasingly understood, their applicability has been reevaluated both as safe antidepressants and for treatments outside of neuromodulation.

While MAO-A and serotonin neurotransmission have long been understood in the brain, more recent research has identified the importance of serotonin signaling throughout the body (El-Merahbi et al., 2015). In fact, the majority of the body’s serotonin is produced in the gut by enterochromaffin cells and is transported by platelets through the bloodstream to recruit immune cells during inflammation and infection (Raghupathi et al., 2013). A recent study identified serotonin signaling as a positive regulator of T cell antitumor activity and reported that MAOIs were able to reduce the anti-inflammatory effects of the TME on T cell function (Wang et al., 2021a). In addition to MAOIs’ ability to increase the stimulation of the TCR signaling pathway through enhanced serotonin receptor agonism, researchers reported that MAOI treatment decreased the alternative, anti-inflammatory polarization of tumor-associated macrophages (TAMs) (Wang et al., 2021b). By depolarizing TAMs, MAOIs serve as an effective TME-engineering therapy that could enhance the efficacy of other ICB therapies. Unfortunately, preliminary studies showed that the systemic administration of the MAOI phenelzine (PLZ) in doses sufficient for T cell and TAM modification also affects the brain and can result in increased aggression not unlike that associated with inactivity of the MAOA gene in mice and humans. (Tiihonen et al., 2015; Chen et al., 2019; Brown et al., 2022). Thus, it is important to evaluate alternative methods for the administration of MAOIs that can decrease neuronal side effects while increasing the efficiency of drug delivery to the tumor.

One potential means of enhancing targeted delivery to the TME is the use of crosslinked multilamellar liposomal vesicles (cMLVs). Liposomes are the most common and best-investigated nanocarriers for drug delivery and have been used across many fields since the 1960s (Joo et al., 2013; Liu et al., 2014). cMLVs are nanoformulated particles that can envelop a therapeutic molecule in a liposomal capsule that improves endocytosis and reduces systemic toxicity (Sercombe et al., 2015). Recently, the field of cancer therapy nanoformulation has seen many translational studies demonstrating successes in improving the TME (Sercombe et al., 2015). The improved stability and endocytosis-conducive nature of liposomal nanocarriers have been shown to greatly reduce systemic toxicity and improve tumor selectivity due to the TME’s enhanced permeability to and retention of liposomal vesicles (Kim et al., 2015; Benadiba and Maor, 2016; Mancini et al., 2018). Our previous publication described the development of a cMLV that has demonstrated improved delivery of the anticancer drug doxorubicin when compared to unilamellar liposomes and free-drug administration (Joo et al., 2013). The crosslinked multi-lamellar structure allowed for a “sustained, controlled drug release profile” and enhanced release kinetics (Joo et al., 2013). There, we provide a detailed investigation into the drug delivery profile of these liposomal vesicles and lay the groundwork for their adaptation for future therapies (Joo et al., 2013).

Thus, this study investigates the therapeutic efficacy of these cMLVs modified to carry an FDA-approved MAOI, phenelzine, which has been proven to have antitumor efficacy, to determine whether there is an improved antitumor response in a mouse syngeneic B16-OVA melanoma model. To evaluate whether nanoformulation affects the distribution of phenelzine to unwanted tissues, we use high performance liquid chromatography (HPLC) to detect the presence of phenelzine in the brain. Additionally, using the resident-intruder aggression test, we seek to determine whether nanoformulation can successfully remediate the aggression-inducing effects of phenelzine by improving the delivery of the drug to the tumor instead of undesired tissues (Hong et al., 2015). The successful development of nanoformulated phenelzine for cancer therapy documented by this study could lead to the establishment of a new therapeutic strategy for repurposing MAOIs as an ICB therapy.

## Methods

### Mice

C57BL/6J (B6) mice were purchased from the Jackson Laboratory (JAX; Bar Harbor). All animals were maintained in the animal facilities at UCLA. Eight-to 12-week-old females were used for all experiments. All animal experiments were approved by the Institutional Animal Care and Use Committee of UCLA.

### Mouse tumor models

The B16-OVA mouse melanoma cell line was provided by P. Wang (University of Southern California, CA, United States). Cells were cultured in D10 adherent cell culture medium made from Dulbecco’s modification of Eagle’s medium (DMEM; catalog no. 10013, Corning) supplemented with 10% fetal bovine serum (FBS; catalog no. F2442, Sigma-Aldrich) and 1% penicillin-streptomycin-glutamine (catalog no. 10378016, Gibco). Cells were then collected and subcutaneously injected into experimental B6 mice (1 × 10^6^ cells per animal) to form solid tumors. Mice received intravenous injection of free phenelzine (10, 20, or 30 mg/kg every 2 days) or nanoformulated phenelzine (cMLV-PLZ) (30 mg/kg every 2 days) to block MAO-A activity. Previous publications established 30 mg/kg as an immunotherapeutic dose of phenelzine in B19-OVA carrying mice, so this dosage was selected for free- and cMLV-PLZ in tumor growth and aggression experiments (Wang et al., 2021a; Wang et al., 2021b). During an experiment, tumor growth was monitored twice per week by measuring tumor size using a Fisherbrand Traceable digital caliper (Thermo Fisher Scientific); tumor area was calculated by multiplying the two axes (length and breadth) of tumors.

### Synthesis of crosslinked multilamellar liposomal vesicles

Liposomes were prepared based on the conventional dehydration-rehydration method. 1.5 μmol of lipids of DOPC, DOPG, and MPB-PE at the molar ratio of the lipid composition of DOPC:DOPG:MPB-PE = 40:10:50, were mixed in chloroform, and the organic solvent in the lipid mixture was evaporated under argon gas and dried under vacuum overnight to form dried thin lipid films. The resultant dried film was hydrated in 10 mm Bis-Tris propane at pH 7.0 containing Phenelzine at a molar ratio of 0.2:1 (drug:lipid), with vigorous vortexing every 10 min for 1 h, and then applied with four cycles of 15-s sonication (Misonix Microson XL 2000, Farmingdale, NY) on ice at 1 min intervals for each cycle. To induce divalent-triggered vesicle fusion, MgCl2 was added to make a final concentration of 10 mm. The resulting multilamellar vesicles were further crosslinked by addition of Dithiothreitol (DTT, Sigma-Aldrich) at a final concentration of 1.5 mm for 1 h at 37°C. The resulting vesicles were collected by centrifugation at 14,000 g (12,300 RPM) for 4 min and then washed twice with PBS. For pegylation of CMLs, the liposomes were further incubated with 1 μmol of 2 kDa mPEG-SH (Laysan Bio Inc, Arab, AL) for 1 h at 37°C. The particles were then centrifuged and washed twice with PBS (Joo et al., 2013). The hydrodynamic size and size distribution of CMLs were measured by dynamic light scattering. CryoEM images were collected using a Tecnai T12 electron microscope (FEI Company) equipped with a Gatan Ultrascan 2k by 2k CCD camera (Joo et al., 2013).

### Pharmacokinetics and presence of phenelzine in brain

HPLC was used to measure brain phenelzine levels as previously described (Tiihonen et al., 2015; Chen et al., 2019). Briefly, brain tissue samples were collected on day 18 post tumor challenge 2 h after final phenelzine injection and snap-frozen using liquid nitrogen. Frozen samples were thawed and homogenized using methanol and acetonitrile by vortexing. Homogenized samples were centrifuged, and supernatants were collected to new tubes and evaporated under a stream of argon. Dried sample pellets were then reconstituted in a solvent with identical composition of the HPLC loading mobile phase. After the extraction of phenelzine, with further purification using an Amicon Ultra 10,000 MWCO centrifugal filter, phenelzine was quantified by reverse phase HPLC using a C18 column in a thermostat with a gradient elution (System Gold 166P detector, Beckman Coulter) (Sercombe et al., 2015). The column was equilibrated by the loading mobile phase with 30 times the column volume before testing. The quantified phenelzine was calculated back and normalized to the mass of the brain tissue.

### Aggression evaluation

A resident-intruder assay was used to examine aggression behavior of a resident mouse in its home cage (Hong et al., 2015). Resident mice in their home cages were transferred to a behavioral testing room containing a customized behavioral chamber equipped with video acquisition capabilities. An unfamiliar (“intruder”) mouse was then introduced into the home cage of the tested resident. The resident and intruder were allowed to interact with each other freely for 15–30 min before the intruder was removed. If fighting resulted in animals sustaining injuries that led to damage to the skin, the interaction was terminated prematurely.

## Results

### Nanoformulation enhances MAOI antitumor therapy

**FIGURE 1:**
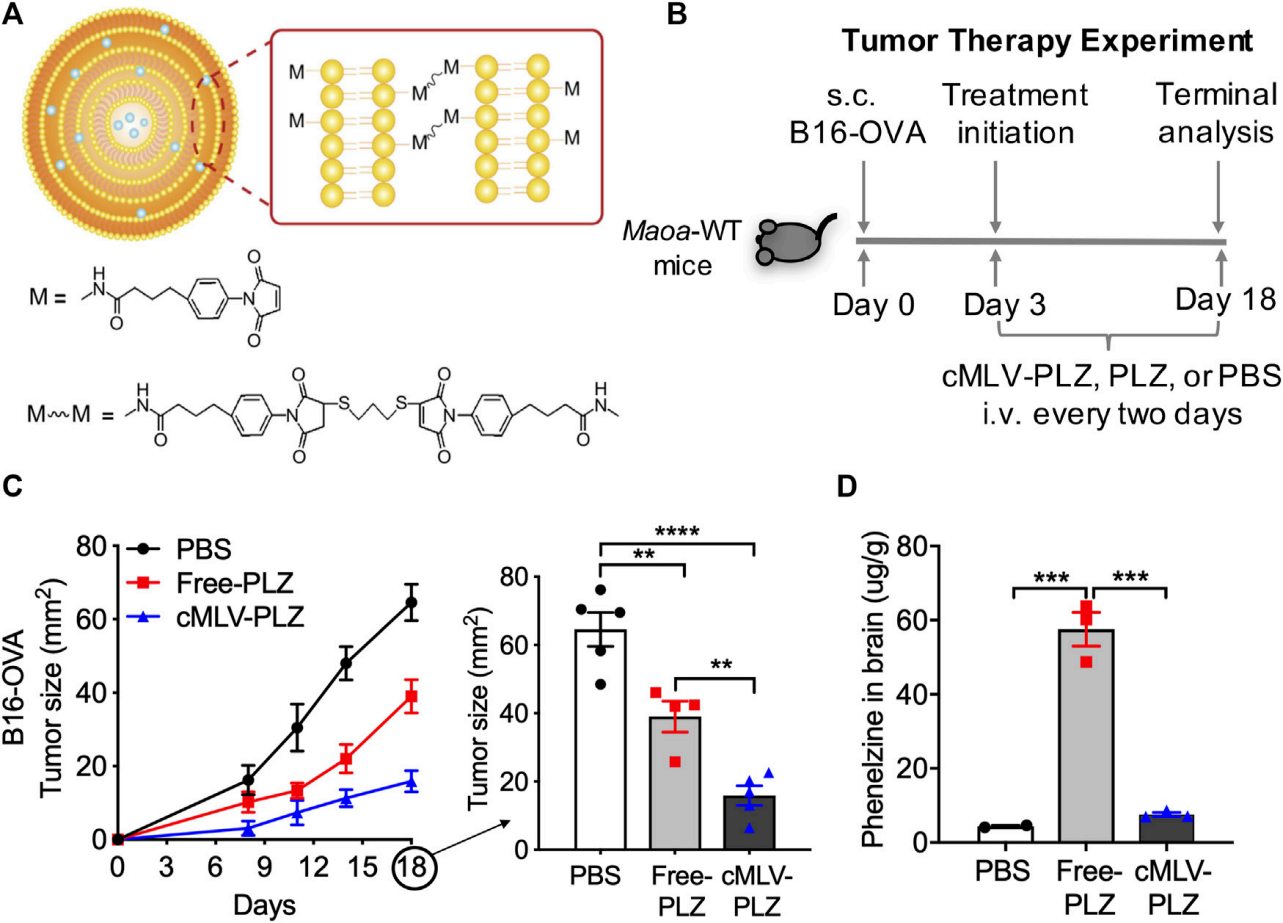
Nanoformulation increases phenelzine-mediated antitumor activity. **(A)** Schematic of cMLV crosslinking design **(B)** Study design for evaluating the cancer therapy potential of cMLV-formulated phenelzine (cMLV-PLZ, 30 mg/kg) in a B16-OVA mouse melanoma model. Free phenelzine (Free-PLZ, 30 mg/kg) was included as a control. **(C)** Tumor growth (*n* = 5) over an 18-days time course study. At day 18, a significant reduction in tumor growth was observed in cMLV-PLZ treated mice compared to free phenelzine of comparable dosage **(D)** Phenelzine (PLZ) measurements in the brain (*n* = 3). At day 18 after tumor challenge, 2 h after final drug injections, mouse experiments were terminated and brain tissues were collected. Measurements were obtained through quantitative reverse phase High Performance Liquid Chromatography on brain tissue samples. Data are presented as the mean ± SEM. **p < 0.01, ***p < 0.001, ****p < 0.0001 by one-way ANOVA.

The establishment of MAOIs as immunostimulatory checkpoint inhibitors raised interest in their repurposing as ICB therapies. Nanoformulation has been the subject of increasing investigation as a promising candidate for on-tumor delivery of cancer therapies due to the stability of liposomal vesicles, their ability to carry drugs of differing hydroaffinities, and their improvement of drug uptake by target cells through traditional and caveolin-mediated endocytosis (Joo et al., 2013; Sercombe et al., 2015). cMLVs are promising liposomal carriers due to the increased stability caused by multi-layered crosslinking of liposomes (Figure 1A) and observed associated improvements in drug delivery (Joo et al., 2013; Zhang et al., 2017). For this study, a crosslinked multilamellar liposomal vesicle was developed to carry phenelzine (cMLV-PLZ), which has been demonstrated to elicit powerful antitumor responses by increasing autocrine serotonin signaling in CD8^+^ T cells, as an alternative delivery system that would limit its systemic administration and improve its delivery to tumor-infiltrating immune cells (Benadiba and Maor, 2016; Wang et al., 2021a). We have demonstrated the drug delivery profile and cMLV quality control in our previous publication detailing the manufacture of these vesicles (Joo et al., 2013). Vesicles were approximately 220 nm in diameter and had a narrow size distribution (polydispersity: 0.101 ± 0.0082) indicating no significant aggregation of particles during crosslinking. The multilamellar structure was further confirmed by cryo-electron microscopy, which exhibited multilayered vesicle formation with thick walls (Joo et al., 2013). *In vitro* encapsulation efficiency was measured at ∼85% for cMLVs (Joo et al., 2013).

In order to evaluate their effects on the anti-tumor efficacy of phenelzine, cMLV-PLZ was administered to B16-OVA tumor-bearing B6 mice at concentrations matching those proven to elicit an antitumor response (Figure 1B) (Wang et al., 2021a; Wang et al., 2021b). The effects on tumor size were compared to that of a control group receiving no treatment and a group receiving unformulated phenelzine. Additionally, since immunotherapeutic doses of phenelzine were observed to induce aggression, the distribution of phenelzine to the brain was quantified in all groups to determine the effect of cMLVs on brain infiltration.

As expected, treatment with free phenelzine resulted in an improved antitumor response when compared to the PBS-treated control group. Much more interestingly, however, was the improved tumor phenotype of the cMLV-PLZ-treated mice when compared to mice receiving free phenelzine. Nanoformulation was strongly correlated with a decrease in tumor size at day 18 when compared to both the PBS control group and free phenelzine group, suggesting improved drug delivery to the TME and activation of tumor infiltrating immune cells (Figure 1C). Additionally, nanoformulation decreased the incidence of free phenelzine in the brain when measured by quantitative HPLC analysis (Figure 1D). While the traditional phenelzine treatment resulted in a large amount of phenelzine infiltration into the brain, cMLV-PLZ resulted in a significantly lower amount of drug infiltration, with levels nearly comparable to the control group. This suggested that nanoformulation successfully reduces the delivery of phenelzine to the brain (Volz and Gleiter, 1998; Benadiba and Maor, 2016; Brown et al., 2022).

### Nanoformulation reduces MAOI-induced aggression

Following the successful nanoformulation of phenelzine and its observed improvement of antitumor activity, the effects of cMLV-PLZ on mouse aggression were examined to determine their impact on side effect reduction. Due to the observed increase in aggressive behavior during preliminary trials and decrease in phenelzine in the brain, a careful catalog of the negative behavioral modifications brought on by MAOI administration was constructed (Volz and Gleiter, 1998). Mice were treated with increasing doses of free phenelzine (10, 20, 30 mg/kg), as well as cMLV-PLZ at the highest dose (30 mg/kg) and a control group treated with phosphate buffered saline (PBS). The extent of the aggression resulting from their treatment was monitored using the resident-intruder aggression test, in which an “intruder” mouse is introduced to an isolated “resident” mouse then observed for aggressive behaviors (Hong et al., 2015). Aggression incidence, intensity, duration, frequency, and latency to first attack were measured for mice in each group.

**FIGURE 2:**
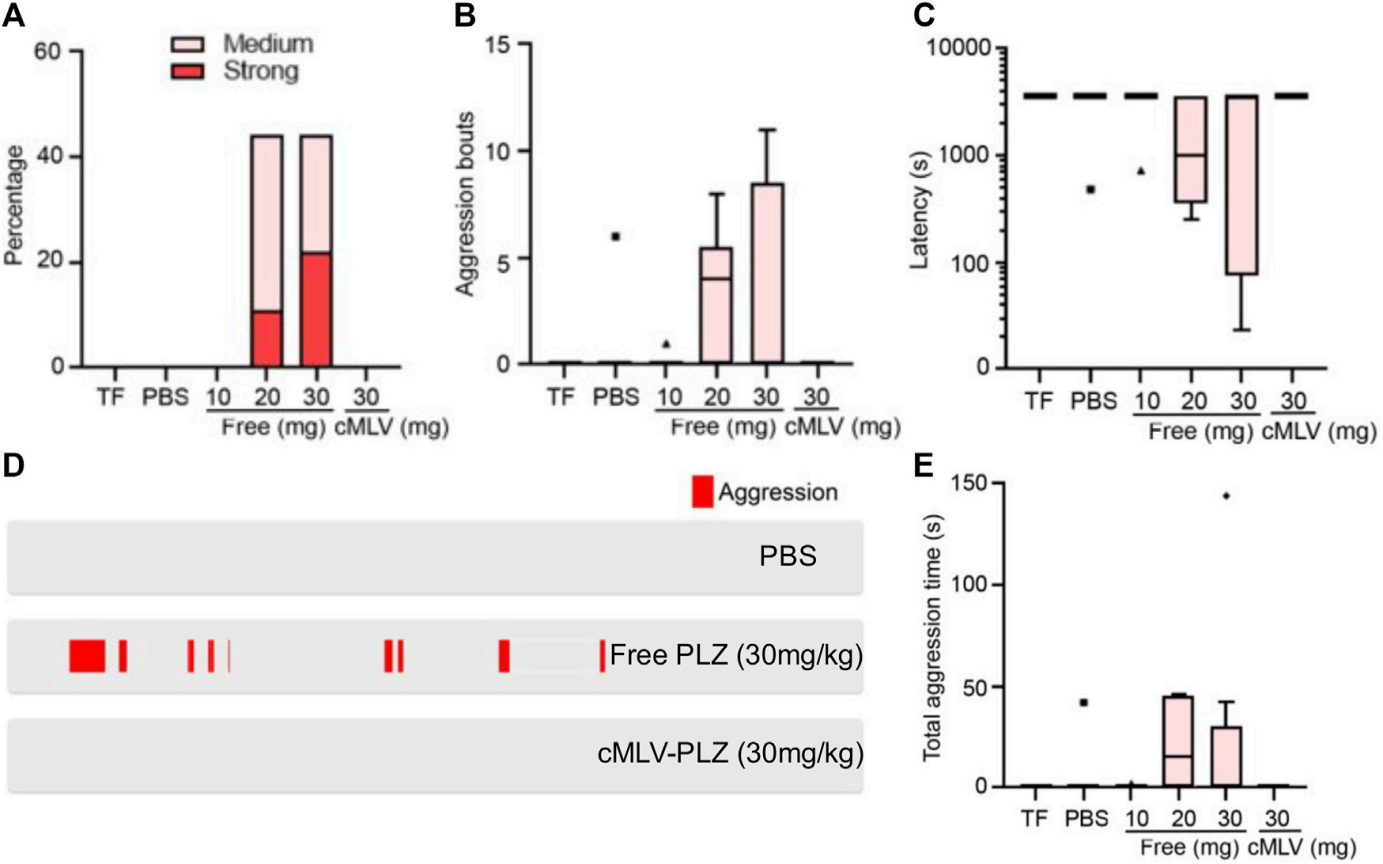
Resident-intruder aggression tests demonstrate decreased aggression in cMLV-PLZ mice. Effects of nanoformulation on MAOI-instigated aggression were investigated in tumor-bearing mice treated with either free or cMLV-formulated phenelzine (TF = Tumor Free; Free-PLZ or cMLV-PLZ; i. v, q3d). N = 5. mg: 30 mg/kg. Mice were treated with indicated does of phenelzine for 2 weeks, followed by behavioral analysis. Mouse aggression levels were recorded using a resident-intruder aggression test. **(A)** Percentage of animals showing medium to strong aggression upon intruder introduction. **(B)** Quantification of aggression bouts per trial across different conditions. **(C)** Quantification of latency to the onset of aggression in each trial across different conditions. **(D)** Representative raster plots showing aggression. **(E)** Quantification of total time the animals engage in aggressive behavior in each trial across different conditions. Data are presented as the mean ± SEM.

Of all mice observed in the control group, only one mouse exhibited low-frequency, low-intensity aggression, setting a low baseline of aggression in untreated mice (Figure 2B). Low-level administration of phenelzine (10 mg/kg) resulted in similar levels of aggression across all trials, indicating that such a low dosage does not have negative behavioral effects in mice (Figures 2A–E). However, because our previous publication demonstrated that higher drug concentrations were used for immune checkpoint blockade (Wang et al., 2021a), trial groups with 20 and 30 mg/kg of administered phenelzine were also included in the study. These groups presented much higher incidences of aggression with increased severity, demonstrating the adverse behavioral side-effects that may result from repurposing the antidepressant as an ICB therapy. Interestingly, cMLV-PLZ-treated mice exhibited aggression levels nearly identical to those of the PBS control and 10 mg/kg free-phenelzine groups across aggression incidence and intensity (Figure 2A), bout number (Figure 2B), bout latency (Figure 2C), and aggression duration (Figure 2E).

The group receiving the highest dosage of free phenelzine (30 mg/kg) saw the greatest increase in percentage of aggressive mice (Figure 2A), severity of aggression (Figure 2A), length of aggressive bouts (Figure 2E), attack latency (Figure 2C), and number of bouts between each “resident” and “intruder” (Figure 2B). While this indicated that drug levels needed for effective ICB therapy could result in serious neurological side effects, the absence of induced aggression in mice treated with an equal dosage of cMLV-PLZ suggests that nanoformulation completely eliminates aggression-inducing side effects of high-dosage phenelzine (Figure 2D). This greatly improves the clinical safety of the drug for ICB therapy by ameliorating the behavioral side effects that decrease therapeutic desirability (Volz and Gleiter, 1998; Mentis et al., 2021).

## Discussion

The successful creation of cMLV-PLZ resulted in improved phenotypic responses with respect to both antitumor activity and aggression-related behavioral side effects. MAOI antitumor efficacy acts through a muti-pronged mechanism in immune regulation and stimulation (Brown et al., 2022). MAO-A inhibition increases stimulatory autocrine serotonin signaling in intratumoral T cells, but it has also been demonstrated to cause decreased immunosuppression through the depolarization of anti-inflammatory TAMs (Wang et al., 2021a; Wang et al., 2021b). Lipid nanocarriers have been incredibly effective in improving the stability of drugs in the serum, with cMLVs being among the most impressive (Joo et al., 2013). Improved stability would greatly increase the per-dose efficacy of therapeutics and could be a major contributor to the observed increase in antitumor activity. Perhaps more interestingly, cMLVs have also been shown to increase endocytosis in previous drug delivery studies, which may be particularly effective in phagocytotic macrophages (Liu et al., 2014; Sercombe et al., 2015). The targeting of macrophages by liposomal carriers may be one mechanism behind the improved therapeutic effects of cMLV-PLZ, though further studies are required to determine the specific pharmacokinetics of this proposed therapy. This may be further enhanced by the permeability and retention effect observed in tumors treated with cMLV drug carriers, increasing the concentration of drug delivered to tumors compared to peripheral tissues (Joo et al., 2013). By directing this therapy towards the immune cells of the TME, nanoformulation can increase the immunostimulatory effects of MAOIs, thus improving the drug’s efficacy.

Examination of brain phenelzine levels found that nanoformulation greatly reduced presence of phenelzine in the brain, and aggression tests saw nearly identical behavioral trends between cMLV-PLZ-treated and control mice. This may be the result of increased distribution to the tumor due to its enhanced permeability to cMLVs, reducing the systemic distribution of phenelzine to other tissues and potentially attenuating the effects of MAO inhibition outside the tumor. These results suggest that nanoformulation is a feasible method for a more targeted delivery of similar drugs with unwanted or unnecessary psychoactive effects. cMLV-PLZ eliminated the aggressive side effects associated with free-phenelzine treatment, suggesting a safer drug profile for cancer patients taking this drug for its ICB properties (Chen et al., 2019). Additionally, while antidepressants can be incredibly effective and beneficial for patients with major depressive disorder, unaffected patients with typical serotonin levels are not recommended behavior-modifying drugs such as MAOIs (Willard et al., 2015). While depression is not uncommon in cancer patients, the nanoformulation of MAOIs used for ICB can prevent the neurological modification of those who would not otherwise be prescribed MAOIs while simultaneously improving their efficacy as an immunotherapy.

While our study of nanoformulation does not address some of the systemic maladies associated with serotonin syndrome, the elimination of high-dose-associated aggression improves both marketability and safety of cMLV-PLZ as an ICB (Volz and Gleiter, 1998; Liu et al., 2014). Although the inhibition of peripheral MAO-A may still result in a reduced systemic ability to degrade tyramine and produce a subsequent tyramine build-up, patients properly educated about the moderate diet-related risks should not be dissuaded from the adoption of this potentially life-saving therapy, especially with the observed efficacy seen in phenelzine/anti-PD-1 combination therapy (Gillman, 2018; Patel and Minn, 2018; Wang et al., 2019; Wang et al., 2021a). While the safety of MAOIs has improved with increased understanding of the drugs and their targets, future investigations into the effects of cMLV encapsulation on systemic side effects could create a comprehensive safety profile for cMLV-PLZ as an anticancer drug. Many cancer therapies have intensely adverse side effects, so the efficacy of MAOIs as a cancer therapy in conjunction with their limited side effects, both of which are enhanced by the nanoformulation described in this study, may make them an appealing alternative (Lavin et al., 1993; Xu and Wang, 2015).

Because the FDA has already approved phenelzine as an antidepressant, the drug can easily be fast-tracked for human application in the cancer setting, especially in light of the improved anti-tumor activity and decreased aggressive side effects observed in this study (Brown et al., 2022). Further studies surrounding the mechanisms behind the systemic pharmacology of nanoformulated MAOI delivery will expedite clinical studies repurposing these drugs to treat cancer patients. Additionally, there are multiple MAOIs that have been developed for the treatment of depression, some of which have been demonstrated to induce antitumor activity in T cells (Wang et al., 2021a). As the narrative surrounding MAOI delivery becomes increasingly comprehensive, future studies should expand their scope to include these drugs.

## Data Availability

The original contributions presented in the study are included in the article, further inquiries can be directed to the corresponding authors.
